# Body Image Concern and Eating Disorder Symptoms Among Elite Icelandic Athletes

**DOI:** 10.3390/ijerph16152728

**Published:** 2019-07-31

**Authors:** Hafrún Kristjánsdóttir, Petra Sigurðardóttir, Sigurlaug Jónsdóttir, Guðlaug Þorsteinsdóttir, Jose Saavedra

**Affiliations:** 1 Physical Activity, Physical Education, Sport and Health Research Centre (PAPESH), Sports Science Department, School of Social Sciences, Reykjavik University, 101 Reykjavik, Iceland; 2 Psychology Department, School of Social Sciences, Reykjavik University, 101 Reykjavik, Iceland; 3 Landspitali—The National University Hospital of Iceland, 101 Reykjavik, Iceland

**Keywords:** Anorexia, bulimia, health, performance, sports

## Abstract

The aim of this study was to analyse body image concerns and symptoms of eating disorders in elite Icelandic athletes according to their sex, and sport practiced. The participants were 755 athletes (24.8 ± 3.5 years in age) who compete at the highest possible level in Iceland. Representing 20 different sports, they were divided into five sports groups. Three questionnaires were used: the Body Shape Questionnaire to assess body image concerns; the Bulimia Test-Revised to assess the main symptoms of bulimia; and the Eating Disorder Examination Questionnaire to identify disordered eating attitudes and behaviours. A chi-squared test was used to analyse differences in prevalence of body image concern and eating disorders, a t-test for the differences between men and women, and a one-way ANOVA to compare the different sports. The main findings were that 17.9% of the athletes presented severe or moderate body image dissatisfaction, and 18.2% (25.3% of the women) were above the clinical cutoff for body image concern. Women’s scores were higher than men’s (whole sample and ball games) in all variables except restraint. These results seem to point to the existence of a real problem that athlete, coaches, doctors, and institutions need to take into account.

## 1. Introduction

It is known that eating disorders, such as anorexia, bulimia nervosa, or binge eating, among others, are complicated to deal with, and that they constitute a public health problem [1]. We shall focus on anorexia and bulimia nervosa. The former is a disorder characterized by deliberate weight loss, induced and sustained by the patient [2] while the latter is a syndrome characterized by repeated bouts of overeating and excessive preoccupation with the control of body weight, leading the patient to adopt extreme measures so as to mitigate the “fattening” effects of ingested food [2]. Related to these two eating disorders is body image dissatisfaction, which can be defined as a negative subjective evaluation of one’s own body. The three, anorexia, bulimia nervosa, and body image dissatisfaction interact with each other [3], and body image dissatisfaction has even been described as a strong predictor of anorexia and bulimia nervosa [4]. In particular, if body image dissatisfaction improves, disordered eating behaviour decreases [5].

The tendency is to think that these eating disorders occur equally in the general population, but there is increasing recognition of their presence in specific populations, including athletes [6]. Indeed, there exists a subclinical eating disorder categorized within anorexia nervosa—“anorexia athletica”. This can be defined as a state of reduced energy intake and reduced body mass despite a high level of physical performance [7]. Athletes embody the concept of physical perfection, and the requirements to meet the standards of weight, eating behaviour, and performance are strict [8]. Certain factors contribute to this sport-specific pressure, e.g., specific judging criteria, weight limits, and tight or revealing uniforms [9]. Also, athletes are constantly under pressure to fit their sport’s stereotypical athletic body. These athletic stereotypes can lead to the risk of body image dissatisfaction among athletes who are struggling to meet the criteria for the ideal body [10]. Moreover, the perfect body to obtain the best performance in a given sport does not always coincide with society’s standard of an aesthetic body. This could lead to the athlete’s even greater dissatisfaction [11]. Body image dissatisfaction is generally considered to be a phenomenon that is primarily experienced by women [12], but the focus is steadily shifting to include men [13]. In a study of 576 German athletes, 59% of the men reported dissatisfaction with their body [14], and even in contact sports like rugby, 46% of elite players have a poor perception of their body image [15]. While there are differences in body dissatisfaction between athletes and non-athletes, the differences between elite and non-elite athletes are unclear [5], and female artistic gymnasts’ body dissatisfaction can change over a single season [4]. Also, a recent study showed that lean-sport athletes had higher body shape concern scores than those of non-lean sports [16].

Athletes who do not fit the ideal body type for their sport may experience more pressure to achieve that ideal body shape, and start to use exaggerated methods which lead to inappropriate dieting, to disordered eating attitudes or behaviours, and to the development of clinical eating disorders [10]. The prevalence of eating disorders varies widely, in the range 6%–45% in female athletes and 0%–19% in male athletes [17]. In a study of 405 elite French athletes (63% male), this prevalence reached 33% [11]. The differences among studies could be due to the heterogeneity of the athletes, to the lack of a clear-cut definition of disordered eating [18], or to the different questionnaires used: the 36-item Bulimia Test-Revised (BULIT-R) [19], the Eating Attitudes Test-26 (EAT-26) [20], the Eating Disorder Examination Questionnaire (EDE-Q) [21] or its short form—the Eating Disorder Examination Questionnaire Short (EDE-QS) [22], among others.

As is the case with body image dissatisfaction, eating disorders have traditionally been seen as occurring more frequently in women than in men, in the regular population. This is indeed what was also found in athletes, with women scoring higher in dietary restraint, eating concern, shape concern, weight concern, and overall score (EDE-Q), although record-holding athletes scored higher on dietary restraint than other athletes [23]. With regard to eating disorders in different sports, they are more prevalent in sports that emphasize specific weights or leanness [24] than in sports where leanness is considered less important for enhanced performance [25]. They are also more prevalent in aesthetic sports than in weight-class sports [7]. However, the differences between individual (track-and-field, swimming, and taekwondo) and team (basketball, handball, volleyball) sports are unclear [23]. Dieting to enhance performance has been reported as being a significant risk factor for eating disorders in aesthetic sports [26] and gymnastics [27]. Likewise, among female upper-secondary school runners, those with an injury scored higher on weight and shape concern and overall score (EDE-Q) than those with no injury [28]. With respect to differences by sex and by sport, among male athletes a greater prevalence of eating disorders is observed in weight-class sports (22%) than in endurance sports (9%) or ball sports (5%), while in women the there is a greater prevalence in aesthetic sports (42%) than in endurance sports (24%) [29].

In recent years, the successes of Icelandic athletes have attracted the interest of the media and researchers [30], but these athletes’ potential problems have been far less studied. Given this context, where eating disorders can be considered a public health problem that affects not only the population in general, but also athletes in particular, the objective of this study was therefore to determine the body image concerns and the symptoms of eating disorders of elite Icelandic athletes according to the sport and their sex.

## 2. Materials and Methods

### 2.1. Participants

Two thousand five hundred Icelandic athletes, competing in 20 different sports at the highest level of competition in Iceland, were invited to participate in the study. The inclusion criteria were: the athletes had to be (i) Icelandic, (ii) at least 18 years old, and (iii) competing at the highest possible level, defined as the top league in ball game sports and the national cup in individual sports. Snowball sampling was used, with 1113 athletes being recruited (44.52% of those invited), and 755 of them completed all the questionnaires (24.8 ± 3.5 years old, range 18 to 35 years, 31.1% men). Thus, 67.83% of the participants who began to respond to the questionnaires completed them (755 out of 1113). The athletes were divided into five sports groups [25]: aesthetic, endurance, weight-class, fitness, and ball sports. The aesthetic sports (*n* = 43, 9.3% men) were artistic gymnastics, group gymnastics, ballet, and modern dance; the endurance sports (*n* = 116, 26.7% men) were long-distance running, swimming, and track and field. The weight-class sports (*n* = 76, 59.2% men) were judo, jiu-jitsu, karate, mixed martial arts, and weightlifting. The fitness sports (*n* = 140, 39.2% men) were crossfit and fitness/body-building. The ball sports (*n* = 380, 27.1% men) were basketball, badminton, football, handball, ice hockey, and volleyball. Although ballet and modern dance cannot be considered sports in any strict sense, they were included in the “aesthetic sports” group to follow the original classification [25].

### 2.2. Instruments

All the final participating athletes filled out three questionnaires in the following order: Body Shape Questionnaire (BSQ), Bulimia Test-Revised (BULIT-R), and Eating Disorder Examination Questionnaire (EDE-Q).

The 34-item Body Shape Questionnaire (BSQ) [31] was used to assess body shape concerns among the athletes. Each item is scored on a 6-point Likert scale with 1 = “Never” and 6 = “Always”. As an example, one item is: *“Have you thought that your thighs, hips, or bottom are too large for the rest of you?”* The total score on BSQ was calculated as the sum of all items with the possible range of overall scores being 34–204 points, where a higher score indicates greater level of body dissatisfaction. The questionnaire has no subscales. The equivalent classification of the total BSQ scores is: less than 80, no concern with shape; 80 to 110, mild concern with shape; 111 to 140, moderate concern with shape; and over 140, marked concern with shape. The psychometric properties of the questionnaire have been tested to be good in both its English [32] and Icelandic [33] versions. The internal consistency of the questionnaire was calculated and appeared to be excellent (Cronbach’s α = 0.975). Its internal consistency by sex was also excellent (men α = 0.962; women α = 0.974). The clinical cutoff score was set at ≥110 points [4].

The 36-item Bulimia Test-Revised (BULIT-R) [19] was used to assess the main symptoms of bulimia (e.g., binge eating, purging, and weight and body shape concerns). BULIT-R is a self-report test in which items are scored on a 5-point Likert scale, with 1 corresponding to the extreme normal response and 5 to the extreme bulimic response. As an example, one item is: *“There are times when I rapidly eat a very large amount of food.”* It can be used to assess changes in bulimia symptoms over time as well as to screen for individuals at risk. The questionnaire has no subscales. The psychometric properties of the questionnaire have been tested to be good in both its English [19] and Icelandic [34] versions. The internal consistency of the questionnaire was calculated and appeared to be excellent (α = 0.932). Its internal consistency by sex was also excellent (men α = 0.906; women α = 0.938). The clinical cutoff score was set at ≥98 points [34].

The Eating Disorder Examination Questionnaire (EDE-Q) [21] was used to identify disordered eating attitudes and behaviours among participants. EDE-Q is a 28-item self-report version of the corresponding semi-structured interview. It provides scores on four subscales—restraint (five items, one example being: *“Have you been deliberately trying to limit the amount of food you eat to influence your shape or weight (whether or not you have succeeded)?”)*, eating concern (five items, one example being: *“Have you had a definite fear of losing control over eating?”),* shape concern (eight items, one example being: *“Have you had a definite desire to have a totally flat stomach?”*), and weight concern (five items, one example being: *“Has your weight influenced how you think about (judge) yourself as a person?”*). Responses are on a 7-point Likert scale, where a higher score reflects a greater eating-related pathology. The overall score is the mean of the four scales. The psychometric properties of the questionnaire have been tested to be good in both its English [35] and Icelandic [36] versions. The internal consistency of the total score and subscales was calculated and appeared to be excellent (total score: α = 0.951; restraint: α = 0.836; eating concern: α = 0.835; shape concern: α = 0.924; weight concern: α = 0.860). Its internal consistency by sex was also excellent (total score: men α = 0.941, women α = 0.953; restraint: men α = 0.816, women α = 0.846; eating concern: men α = 0.791, women α = 0.842; shape concern: men α = 0.912, women α = 0.923; weight concern: men α = 0.812, women α = 0.867). The clinical cutoff score was set at ≥4 points [37].

### 2.3. Procedure

The data were acquired through an online survey from October 2015 to February 2016. In the snowball sampling procedure, the researchers contacted athletes, teams, coaches, and sports associations asking them either to forward a request for participation in the online survey to athletes that met the inclusion criteria or to provide the researchers with contact information about participants in the corresponding particular sport. Generally, these interactions were via Facebook or email, so that the blank online survey could be included simultaneously. The researchers informed the contacts about the purpose of the study. Participants were told that the main objectives of the study were to evaluate eating disorder symptoms and body image among Icelandic athletes. Prior to completing the survey, the participants read the consent forms and then indicated their agreement to take part in the study. They were made aware that their answers were anonymous and that they had the option to drop out at any time while responding to the online survey. No time limit was set for responding to the three questionnaires. The study was approved by the Icelandic Data Protection Authority, and permission for the study was obtained from the National Bioethics Committee in (no.: VSN-15-078). Participation in the study was voluntary, and the participants received no compensation of any kind.

### 2.4. Data Analysis

Cronbach’s alpha (α) was calculated to determine the internal consistency of each questionnaire. All the variables satisfied the tests of homoskedasticity (Levene’s homogeneity test) and normality (Kolmogorov–Smirnov test). The basic descriptive statistics (mean and standard deviation) were calculated. An independent sample t-test was used to examine differences by sex, and a chi-squared test to compare the categorical frequencies. In the analysis of body image dissatisfaction, the z-score, chi-squared, and *p*-value [38] were calculated for each sport. Type I errors were minimized using the Bonferroni correction (Bonferroni correction = 0.05/number of subsamples) [39]. Since in this case there were four levels and five sports groups, the Bonferroni correction = 0.05/20 so that only variables whose p-value was less than 0.0025 were considered. In each of the three questionnaires, the clinical cutoff points were used to calculate the prevalence. Finally, a one-way analysis of variance (ANOVA) was used to examine the differences in means between sports groups on each questionnaire (BSQ, BULIT-R, and EDE-Q), followed by a Games–Howell post hoc procedure for unequal variances (as determined by Levene’s test) or otherwise a Bonferroni post hoc comparison. Again, the Bonferroni correction was applied (Bonferroni correction = 0.05/number of subsamples) [39]. Now, as there were six groups of sports (Bonferroni correction = 0.05/6), only variables whose *p*-value was less than 0.008 were considered. Finally, Pearson simple correlation coefficients between the totals of each questionnaire (BSQ, BULIT-R, and EDE-Q) were calculated for both the whole sample and for men and women separately. The values of this statistic were assigned linguistic labels: 0.1 small, 0.3 moderate, 0.5 large, 0.7 very large, and 0.9 nearly perfect [40]. For these analyses, the Statistical Package for the Social Sciences (SPSS) version 22 (IBM, Armonk, NY, USA) was used.

## 3. Results

Table 1 lists the frequencies of athletes according to their level of dissatisfaction about their own bodies (the chi-squared and *p*-values are also given). The prevalence of the athletes in the whole sample who reported severe or moderate dissatisfaction was 17.9%, and 61.6% were categorized as having no dissatisfaction about their body. In the whole sample, a greater percentage (16.3%) of aesthetic sports athletes were classified as having severe dissatisfaction, than in the other sports groups. Overall, the endurance athletes reported fewer symptoms of body dissatisfaction than the other sports groups (76.7%).

Table 2 lists the prevalence of athletes scoring above the clinical cutoff score. In BSQ, 25.3% of the women athletes were above the cutoff score of 110 compared to 3.9% of the men athletes. In BULIT-R, the equivalent proportions were 2.7% of the women and 1.8% of the men athletes, and in the overall scale of EDE-Q, they were 10.7% of the women and 6.8% of the men.

Table 3 presents the mean scores and their standard deviations for the whole sample, and for the men and women athletes in each sports group and for each questionnaire. Comparison between sports showed that aesthetic sports (whole sample) scored higher in BSQ than the fitness and weight-class sports. There were no differences in any sport in BUILT-R. Restraint (EDE-Q) was greater in fitness than in ball sports (whole sample). With regard to the differences between the sexes, for the whole sample, women scored higher than men on all the questionnaires except for the restraint subscale of EDE-Q. Their scores on BSQ were also higher than the men’s in all the sports groups. On the eating concern subscale of EDE-Q, again women had higher scores than the men in endurance, fitness, and ball sports. The results were similar on the shape concern subscale (fitness and ball sports).

Table 4 lists the correlation coefficients between the final score on each of the three questionnaires, both for the entire sample and for men and women separately. They all correspond to very large correlations [40] between the questionnaires (r ≥ 0.724, *p* < 0.01).

## 4. Discussion

This study has examined the body image concerns and eating disorder symptoms of elite Icelandic athletes in 20 different sports. The main findings were that 17.9% of the athletes in the study presented severe or moderate body image dissatisfaction, with 18.2% being above the clinical cutoff for body image concern, 2.4% above the cutoff for bulimia, and 9.5% above the cutoff for eating disorder symptoms. With regard to the differences by sex (whole sample), the women’s values were higher for all the variables studied except restraint (EDE-Q). This was also the case in each group of sports for BSQ.

To the best of our knowledge, this has been the first study conducted on a large sample of elite Icelandic athletes in which comparisons by sex and by group of sports allow general conclusions to be drawn that are able to be extrapolated to other athletes and countries with similar characteristics. The choice of Icelandic athletes for this study was for our interest in analysing the potential problems that may arise in sports men and women in a country that is so small population-wise relative to its success internationally [30]. The results referring to body image concerns are very worrisome. Of the whole sample, 17.9% stated that they have severe or moderate body image dissatisfaction, with this proportion reaching 39.5% in aesthetic sports (Table 1). It should be borne in mind, however, that 90.7% of the sample in this type of sport were women, which could have influenced the results. The whole sample values are similar to those reported in previous studies conducted in men and women jointly [41] or in men only [3]. The prevalence surpassing the clinical cutoff score for body image concerns was 25.3% for the women and 18.2% for the whole sample (Table 2). These results are coherent with previous findings reporting body image concerns to be closely related to disordered eating behaviours [42]. In this sense, a study of Brazilian adolescent male athletes in 18 different sports found an acceptable age-adjusted correlation (r = 0.28, *p* < 0.01) between body image concerns and eating disorders [43]. Similarly, in female artistic gymnasts, body image concern has been found to be the strongest predictor of eating disorders (0.69 ≤ r ≤ 0.87; *p* < 0.01) regardless of the season in which the evaluation is made [4]. We also found that the prevalence of surpassing the clinical eating disorder cutoff score was high in both the whole sample (9.5%) and in women alone (10.7%), once again showing body image dissatisfaction to be a key factor in understanding the course of eating disorder symptoms. However, it should be noted that the prevalence of eating disorders is greater in elite than non-elite female athletes [44]. Similarly, the prevalence of eating disorders in this study was greater than that found in the general population [45]. This may be due to pressure from the sport’s environment to conform to an ideal body shape in order to improve performance [46]. For men however, not only is the prevalence of eating disorders (6.8%) lower than in women, but neither does it seem to be related to the athletes’ competitive level [47].

The body image concern score (BSQ) was higher in women than in men in both the whole sample and in each of the groups of the sports analysed (Table 3). This is in line with previous findings for Norwegian [29] and French [48] athletes. The values were higher for aesthetic sports than for endurance, fitness, and weight-class sports. This is perhaps because body shape is less determinant in the latter class of sports. Indeed, previous studies suggest the same type of difference between aesthetic and weight-class sports, with there being research connecting minimal percentage body fat with patterns of judging [49]. With regard to bulimia (BULIT-R), the women’s values were greater than the men’s in the whole sample and in ball sports, but there were no differences between any two groups of sports. Although the data collection of the present study was carried out over two months, this period of time would seem not to influence the results since a study on swimmers and gymnasts found no changes in the results of its questionnaire between the beginning and the end of the season [50]. One must add that, although bulimia is less prevalent in men than in women, it can be predicted in male collegiate athletes (R^2^ = 0.48) through dietary restraint, negative affect, and drive for muscularity [51]. Finally, with respect to eating disorders (EDE-Q), women scored higher than men overall and on all the subscales except restraint (EDE-Q) for both the whole sample and the ball sports. These results are consistent with those of previous studies showing that female athletes are at a greater risk of suffering eating disorders [17,23,29,52,53]. There is also evidence that eating disorders are related to ethnicity, sport, and self-esteem [54]. The male athletes scored higher on overall EDE-Q than collegiate [55] or Portuguese local [23] athletes. This may have been because the present study’s sample comprised elite athletes. It was surprising that the endurance sports athletes did not score higher on EDE-Q since those sports are generally linked to leanness as an important factor in optimizing performance, and therefore to the competing athletes being at high risk of eating disorder symptoms [29]. Nevertheless, for the whole sample without distinguishing the sports, the overall score was in or near the 90th percentile in men and the 70th percentile in women relative to the reference values [55]. Finally, there were clear correlations between the three questionnaires for the entire sample as well as for men and women separately (Table 4). In this sense, body image dissatisfaction is a strong predictor of bulimia nervosa [4] and is inversely related to disordered eating behaviour [5]. In sum, body image dissatisfaction, anorexia nervosa, and bulimia nervosa are related to each other [3].

The present study has a number of limitations. First, its cross-sectional nature inhibits us from drawing causal conclusions from the results. However, the size (*n* = 755) and level (the highest level of Icelandic athletes) of the sample suggests that this bias can be regarded as minor, even though the response rate was low (44.52% of those invited). Second, the athletes were examined through self-report data. So there is a chance that self-report bias influenced the results in the sense that the respondents might tend to under-report their symptoms, among other reasons, to protect themselves as they are fearful of negative reactions from family, coaches, or teammates [56]. As is recommended [23], the anonymity of the questionnaires was guaranteed at all times with the intention of ensuring the greatest possible sincerity in the study. Nonetheless, this fact is inherent to all studies that use questionnaires, and must be recognized and accounted for by such studies’ authors. Third, the questionnaires used in the present study identify symptoms of eating disorders and body image disturbances, but they do not provide a clear-cut diagnosis of those disorders. Clinical interviews are needed to obtain more exact prevalence figures [29]. The results do, however, reflect abnormal thinking and behaviours among athletes that should be taken seriously.

## 5. Conclusions

In summary, 17.3% of the study sample analysed presented severe or moderate body image concern, reaching 39.4% in aesthetic sports. The prevalence of body image concern surpassing the clinical cutoff score was 25.3%. In general, for the whole sample, women scored higher than men on almost all the subscales of all the questionnaires. This was also the case in ball sports, as documented for the first time, suggesting that these sports, which until now did not seem to present differences between men and women, also need to be focused on. The results have shown that a real problem exists, and that it must be taken into account by athletes, coaches, doctors, and institutions. In particular, athletes are more likely to seek help from doctors because of decreased performance rather than because of symptoms of clinical problems like eating disorders. Increased awareness will therefore not only help address this growing public health issue, but also make early intervention more likely, and thus help the affected athletes avoid suffering more harm.

## Figures and Tables

**Table 1:** Frequency distribution of body image dissatisfaction (% in parentheses), z-score, chi-squared, and *p*-value.

Measure	Total (*n* = 755)	Aesthetic Sports (*n* = 43)	Ball Sports (*n* = 380)	Endurance Sports (*n* = 116)	Fitness Sports (*n* =140)	Weight Class Sports (*n* = 76)	χ^2^	*p*
Severe body dissatisfaction							34.238	<0.001
*n* (%)	55 (7.3)	7 (16.3)	30 (7.9)	6 (5.2)	8 (5.7)	4 (5.2)		
z-score		2.40	0.10	−0.50	−0.70	−0.70		
χ^2^		5.76	0.01	0.25	0.49	0.49		
*p*		0.016	0.920	0.617	0.484	0.483		
Moderate body dissatisfaction								
*n* (%)	80 (10.6)	10 (23.2)	46 (12.1)	5 (4.3)	12 (8.6)	7 (9.2)		
z-score		1.80	1.30	−2.10	−0.80	−0.40		
χ^2^		3.34	1.69	4.41	0.64	0.16		
*p*		0.071	0.194	0.036	0.424	0.689		
Mild dissatisfaction								
*n* (%)	155 (20.5)	8 (18.6)	92 (24.2)	16 (13.8)	28 (20.0)	11 (14.5)		
z-score		−0.30	2.80	−2.00	0.10	−2.00		
χ^2^		0.09	7.84	4.00	0.01	4.00		
*p*		0.764	0.005	0.046	0.920	0.046		
No dissatisfaction								
*n* (%)	465 (61.6)	18 (41.9)	212 (55.8)	89 (76.7)	92 (65.7)	54 (71.1)		
z-score		−2.80	−3.20	3.30	0.90	2.20		
χ^2^		7.84	10.24	10.89	0.81	4.84		
*p*		0.005	0.001	0.001	0.368	0.028		

**Table 2:** The prevalence of athletes scoring above the clinical cutoff score.

Measures	BSQ *n* (%)	BULIT-R *n* (%)	EDE-Q *n* (%)
Whole sample	138 (18.3)	18 (2.4)	72 (9.5)
Men	9 (3.8)	4 (1.7)	16 (6.8)
Women	131 (25.3)	14 (2.7)	55 (10.6)

BSQ: The Body Shape Questionnaire; BULIT-R: Bulimia Test-Revised; EDE-Q: Eating Disorder Examination Questionnaire.

**Table 3:** Values for each of the questionnaires (total and subscales). Mean, standard deviation, and t-test values for the differences between men and women, and one-way analysis of variance (ANOVA) with the Games–Howell post hoc test for differences between sports groups.

Questionnaire		Whole Sample M ± SD	Aesthetic (a) M ± SD	Ball Games (b) M ± SD	Endurance (c) M ± SD	Fitness (d) M ± SD	Weight Class (e) M ± SD	F	Differences	
BSQ		Whole sample	77.28 ± 34.16	95.81 ± 39.31	79.90 ± 34.13	67.68 ± 32.27	75.96 ± 31.80	70.75 ± 31.78	5.70 ***	a > c, d, e; b > c
		Men	59.08 ± 24.47	54.60 ± 16.70	59.25 ± 24.68	53.87 ± 25.33	61.83 ± 23.95	62.77 ± 23.52	0.85	-
		Women	86.41 ± 34.59	101.24 ± 38.26	87.58 ± 34.00	79.55 ± 33.01	83.32 ± 33.04	81.39 ± 38.04	2.28	-
		t (men vs. women)	−12.73 ***	4.25 *	17.26 ***	11.05 ***	9.09 **	9.91 **		
BULIT-R		Whole sample	47.47 ± 17.64	50.94 ± 14.63	47.03 ± 17.07	46.23 ± 19.40	47.96 ± 19.04	48.77 ± 17.51	0.53	-
		Men	45.31 ± 14.99	42.40 ± 4.51	43.51 ± 13.98	44.23 ± 16.92	47.14 ± 16.48	48.05 ± 14.13	1.40	-
		Women	48.52 ±18.71	55.24 ± 15.23	48.31 ± 17.91	47.80 ± 21.17	48.37 ± 20.27	49.68 ± 21.24	0.68	-
		t (men vs. women)	−2.41 ***	−3.57	8.03**	3.27	1.18	2.98		
EDE-Q	Total	Whole sample	2.17 ± 1.16	2.60 ± 1.17	2.13 ± 1.14	1.97 ± 1.08	2.40 ± 1.22	2.22 ± 1.27	2.19	-
		Men	1.85 ± 1.01	2.06 ± 1.31	1.73 ± 1.00	1.65 ± 8.86	2.15 ± 0.94	2.22 ± 1.18	1.58	-
		Women	2.31 ± 1.20	2.67 ± 1.17	2.26 ± 1.16	2.23 ± 1.17	2.50 ± 1.32	2.21 ± 1.38	1.17	-
		t (men vs. women)	−4.36 **	0.03	4.24 **	3.33	2.88	0.01		
	Restraint	Whole sample	1.99 ± 1.28	2.29 ± 1.40	1.81 ± 1.14	2.00 ± 1.37	2.47 ± 1.37	2.02 ± 1.28	4.01 ***	b < d
		Men	1.94 ± 1.29	2.10 ± 1.71	1.62 ± 1.00	1.82 ± 1.30	2.71 ± 1.44	2.10 ± 1.24	4.03	-
		Women	2.00 ± 1.27	2.31 ± 1.39	1.87 ± 1.17	2.13 ± 1.42	2.36 ± 1.35	1.93 ± 1.33	2.23	-
		t (men vs. women)	−0.56	0.05	0.08	1.22	1.45	−0.01		
	Eating concern	Whole sample	1.52 ± 0.94	1.64 ± 0.84	1.44 ± 0.80	1.62 ± 1.19	1.63 ± 1.01	1.63 ± 1.07	1.38	-
		Men	1.35 ± 0.74	1.35 ± 0.57	1.22 ± 0.58	1.45 ± 0.94	1.31 ± 0.64	1.58 ± 0.85	1.18	-
		Women	1.60 ± 1.00	1.68 ± 0.87	1.51 ± 0.85	1.76 ± 1.35	1.75 ± 1.10	1.69 ± 1.27	1.08	-
		t (men vs. women)	−3.26 ***	0.35	13.27 ***	3.96 *	7.40 **	0.50		
	Shape concern	Whole sample	2.73± 1.59	3.49 ± 1.60	2.80 ± 1.61	1.25 ± 1.32	2.90 ± 1.62	2.58 ± 1.65	6.72	
		Men	2.10 ± 1.37	2.04 ± 1.30	2.05 ± 1.39	1.68 ± 1.03	2.51 ± 1.31	2.52 ± 1.61	2.03	-
		Women	3.02 ± 1.61	3.65 ± 1.57	3.05 ± 1.61	2.73 ± 1.35	3.07 ± 1.73	2.66 ± 1.73	1.68	-
		t (men vs. women)	−6.73 ***	0.37	6.64 *	2.20	4.99 *	0.15		
	Weight concern	Whole sample	2.43 ± 1.50	2.94 ± 1.50	2.42 ± 1.50	2.28 ± 1.40	2.56 ± 1.59	2.37 ± 1.59		
		Men	1.92 ± 1.21	2.10 ± 1.21	1.82 ± 1.17	1.80 ± 1.18	2.11 ± 1.26	2.24 ± 1.33	0.97	-
		Women	2.66 ± 1.57	3.07 ± 1.52	2.63 ± 1.54	2.65 ± 1.46	2.76 ± 1.69	2.52 ± 1.86	0.87	-
		t (men vs. women)	−5.98 ***	0.86	13.03 ***	2.67	2.55	2.85		

*p* < 0.05; * *p* < 0.01; *** *p* < 0.001.

**Table 4:** Pearson simple correlation coefficients between the totals of each questionnaire both for the entire sample and for the men and women separately. *p* < 0.01 for all the correlations.

	Whole Sample			Men			Women		
	BSQ	BULIT-R	EDE-Q	BSQ	BULIT-R	EDE-Q	BSQ	BULIT-R	EDE-Q
BSQ	-			-			-		
BULIT-R	0.724	-		0.777	-		0.742	-	
EDE-Q	0.883	0.801	-	0.889	0.813	-	0.887	0.810	-

BSQ: The Body Shape Questionnaire; BULIT-R: Bulimia Test-Revised; EDE-Q: Eating Disorder Examination Questionnaire.
